# Effects of dry-wet cycles on nitrous oxide emissions in freshwater sediments: a synthesis

**DOI:** 10.7717/peerj.10767

**Published:** 2021-02-12

**Authors:** Renata Pinto, Gabriele Weigelhofer, António Guerreiro Brito, Thomas Hein

**Affiliations:** 1 Instituto Superior de Agronomia, University of Lisbon, LEAF - Linking Landscape, Environment, Agriculture and Food, Lisbon, Portugal; 2 University of Natural Resources and Life Sciences, Institute of Hydrobiology and Aquatic Ecosystem Management, Vienna, Austria; 3 WasserCluster Lunz GmbH –Inter-university Center for Aquatic Ecosystem Research, Lunz am See, Austria

**Keywords:** Aquatic-terrestrial interface, Intermittent, Inland waters, Lotic, Lentic, Drought, Flooding

## Abstract

**Background:**

Sediments frequently exposed to dry-wet cycles are potential biogeochemical hotspots for greenhouse gas (GHG) emissions during dry, wet and transitional phases. While the effects of drying and rewetting on carbon fluxes have been studied extensively in terrestrial and aquatic systems, less is known about the effects of dry-wet cycles on N_2_O emissions from aquatic systems. As a notable part of lotic systems are temporary, and small lentic systems can substantially contribute to GHG emissions, dry-wet cycles in these ecosystems can play a major role on N_2_O emissions.

**Methodology:**

This study compiles literature focusing on the effects of drying, rewetting, flooding, and water level fluctuations on N_2_O emissions and related biogeochemical processes in sediments of lentic and lotic ecosystems.

**Results:**

N_2_O pulses were observed following sediment drying and rewetting events. Moreover, exposed sediments during dry phases can be active spots for N_2_O emissions. The general mechanisms behind N_2_O emissions during dry-wet cycles are comparable to those of soils and are mainly related to physical mechanisms and enhanced microbial processing in lotic and lentic systems. Physical processes driving N_2_O emissions are mainly regulated by water fluctuations in the sediment. The period of enhanced microbial activity is driven by increased nutrient availability. Higher processing rates and N_2_O fluxes have been mainly observed when nitrification and denitrification are coupled, under conditions largely determined by O_2_ availability.

**Conclusions:**

The studies evidence the driving role of dry-wet cycles leading to temporarily high N_2_O emissions in sediments from a wide array of aquatic habitats. Peak fluxes appear to be of short duration, however, their relevance for global emission estimates as well as N_2_O emissions from dry inland waters has not been quantified. Future research should address the temporal development during drying-rewetting phases in more detail, capturing rapid flux changes at early stages, and further explore the functional impacts of the frequency and intensity of dry-wet cycles.

## Introduction

Climate change is expected to alter the frequency and intensity of extreme events. Climate change scenarios predict an increase in both prolonged drought periods and heavy rainfall events, intensifying seasonal runoff and flood risk even in areas where precipitation is expected to decline (Seneviratne et al., 2012). This will increase the magnitude and frequency of dry-wet cycles in aquatic ecosystems in the future. The global spatio-temporal expansion of lentic and lotic water bodies that fall periodically dry in response to climate change, land-use change and water abstraction, has raised further awareness to the effects of dry-wet cycles on biotic communities and biogeochemical processes in inland waters (Datry, Bonada & Boulton, 2017).

Temporary rivers and streams, which are characterized by a periodic cease in water flow, constitute over 50% of the length of the global river network (Datry, Larned & Tockner, 2014). Furthermore, ponds, lakes and reservoirs are known to experience occasional, recurrent or permanent drying. These temporary systems can experience alternating dry and wet conditions of different magnitude, duration, and predictability (Datry, Bonada & Boulton, 2017). Furthermore, specific areas present in all river systems are recurrently exposed to dry-wet cycles, such as the parafluvial zone, which is flooded during high flows and falls dry during low flows. Similarly, water level fluctuation effects are greatest in shallow water and littoral zones of lentic systems, where small drops in water levels can result in large areas of air exposed sediments and vice-versa (Leira & Cantonati, 2008). These systems are also increasingly influenced by climate change in such a way that increases in water level fluctuation magnitude are consistent with hydrological extreme events (Gownaris et al., 2018).

Temporary systems have been less studied than perennial systems, not least because of their complexity. Leigh et al. (2015) and Arce et al. (2019) recently highlighted knowledge gaps in these ecosystems, namely the greater exploration of aquatic-terrestrial linkages and dry-wet cycling. Research suggests that sediments in temporary systems, as well as in water drawdown areas in both lotic and lentic systems, are potential biogeochemical hotspots during dry, wet and transitional phases (McClain et al., 2003; Amalfitano et al., 2008; Gallo et al., 2014; Von Schiller et al., 2017; Von Schiller et al., 2019). Until recently, dry habitats have been viewed as bio-geochemically inert, but studies have shown the importance of including dry phases in global emission budgets (Steward et al., 2012; Catalan et al., 2014; Von Schiller et al., 2014; Gómez-Gener et al., 2015; Gómez-Gener et al., 2016; Kosten et al., 2018; Obrador et al., 2018; Arce et al., 2019; Marcé et al., 2019).

It is well established that drying and rewetting events affect microbial communities and function, and biogeochemical processes in sediments (Baldwin & Mitchell, 2000; Amalfitano et al., 2008; Marxsen, Zoppini & Wilczeck, 2010; Larned, Datry & Robinson, 2007; Zoppini & Marxsen, 2010; Pohlon, Fandino & Marxsen, 2013). Furthermore, the hydrological history, i.e., the frequency, intensity and duration of these events also affect microbial responses to dry-wet cycles (Langhans & Tockner, 2006; Larned, Datry & Robinson, 2007; Foulquier et al., 2015). While the effect of dry-wet cycles on nitrous oxide (N_2_O) emissions has been extensively studied in soils (Borken & Matzner, 2009; Kim et al., 2012; Congreves et al., 2018), it has received less attention in sediments of aquatic systems. In addition, several studies addressing biogeochemical rates in sediments exposed to dry-wet cycles have focused on carbon (C) fluxes (Gómez-Gener et al., 2015; Gómez-Gener et al., 2016; Kosten et al., 2018; Obrador et al., 2018; Marcé et al., 2019; Von Schiller et al., 2014; Von Schiller et al., 2019), with fewer studies focusing on N_2_O emissions (e.g.,  Arce et al., 2018; Pinto et al., 2020). Sediments and soils differ in major physical attributes, such as structure, but are comparable in terms of microbial communities and biogeochemical processes, sharing key drivers of biogeochemical processes and responses to environmental change (Arce et al., 2019).

This synthesis compiles literature focusing on N_2_O emissions from freshwater sediments experiencing alternating dry-wet conditions, which has only been reviewed for other GHGs in inland waters so far (Marcé et al., 2019). The aim is to provide an overview of the current knowledge regarding the effects of dry-wet cycles on N_2_O emissions in sediments, highlight directions for future research and stimulate further interest in a currently understudied topic. In ‘Summary of the main microbial sources of N_2_O’, the main microbial pathways leading to N_2_O production are summarized. ‘Effects of dry-wet cycles on N_2_O emissions in freshwater sediments’ provides an overview of the studies focusing on the effects of dry-wet cycles on N_2_O emissions, in freshwater sediments. This section is divided in three subsections: effects of drying (‘Effects of drying on N_2_O emissions in freshwater sediment’), effects of rewetting by rainfall events (‘Effects of rainfall events on N_2_O emissions in freshwater sediments’) and effects of flooding and water level fluctuations (‘Effects of flooding and water level fluctuations on N_2_O emissions in freshwater sediments: 345 aquatic-terrestrial transition zones’) on N_2_O emissions. The effects of rewetting were separated in rainfall events, and flooding and water level fluctuations to make a clearer distinction between these rewetting events, as they differ in their magnitude and frequency. Each subsection describes how the physical mechanisms and N processes are affected during each phase, followed by the effects on N_2_O emissions. ‘Effects of dry-wet cycles on microbial community composition and function’ briefly addresses the effects of dry-wet cycles on microbial community and function. Finally, concluding remarks and research gaps are highlighted. Exploring the relevance of N_2_O emissions from sediments prone to dry-wet cycles and considering emissions from temporary systems and water drawdown areas is important to improve global estimates of N_2_O fluxes from inland waters.

### Survey methodology

A search for relevant published studies focusing on dry-wet cycling and N_2_O emissions from sediments was performed using the literature database search engines Web of Science and Google Scholar, with a combination of search terms: dry* wet* OR dry* rewet* OR dry* flood* OR drought AND “nitrous oxide” OR ”N_2_O” AND sediment*. The asterisk (*) enables key word variations. The reference lists within the selected papers was also examined. The initial screening of titles and abstracts excluded papers considering N_2_O emissions from soils exclusively, and from sediments other than from freshwater ecosystems. The following studies were excluded from this synthesis: soils (including wetland soils and peatlands), saline or brackish water environments, aquaculture ponds, and rice/paddy field studies. The studies include both field and laboratory observations. Rewetting of dry sediments include events caused by rainfall (natural or simulated), flooding and water table fluctuations. Drying events include water drainage from the sediment and sediment desiccation. N_2_O emission peaks were considered as a direct response to sediment drying and rewetting events. Last database search: 28 of September 2020.

## Summary of the main microbial sources of N_**2**_O

**Figure 1:**
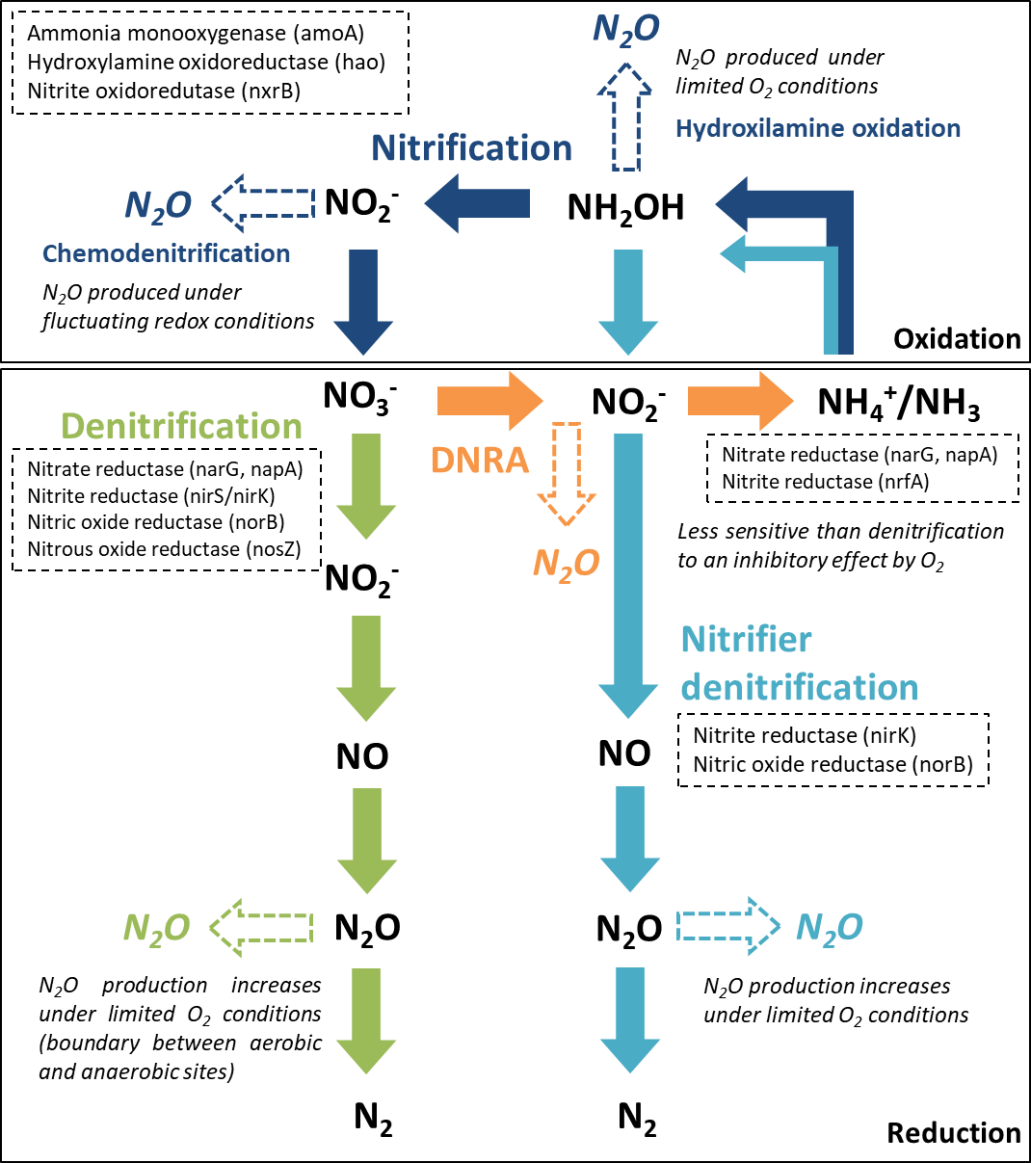
Major nitrogen cycle processes that produce N_2_O. Dashed arrows indicate the steps were N_2_O emissions may occur, along each reaction pathway (adapted from Giles et al., 2012, CC BY 3.0 SA). The enzymes and encoding genes of each process are indicated in the dashed boxes. A reference is made to the O_2_ conditions promoting N_2_O production.

From the major processes involved in the nitrogen cycle in soil and sediments, four microbially mediated reaction pathways can contribute to N_2_O emissions (Baggs, 2011; Quick et al., 2019): nitrification and nitrifier denitrification in oxic environments and denitrification and dissimilatory nitrate reduction to ammonium (DNRA) in suboxic and anoxic environments. The reactive nitrogen species are oxidized or reduced through a sequence of electron transfer steps, promoted by enzymatic reaction pathways. In all of these pathways, N_2_O is produced as an intermediate reaction product (Fig. 1). In aquatic ecosystems, denitrification is regarded as the predominant source of N_2_O, and nitrifier-denitrification is likely more significant than nitrification (Quick et al., 2019). These processes can co-occur over a broad range of oxygen (O_2_)/redox and moisture content (MC) conditions, within oxic/anoxic microsites in sediments (Jørgensen and Revsbech, 1985; Seitzinger et al., 2006).

Denitrification, a facultative anaerobic process, is the reduction of nitrate (NO${}_{3}^{-}$) or nitrite (NO${}_{2}^{-}$) to N_2_O and di-nitrogen (N_2_) performed by heterotrophic bacteria (denitrifiers). Denitrifying microorganisms also include ammonia-oxidizing chemolithotrophic bacteria, which reduce NO${}_{2}^{-}$ to N_2_O aerobically, archaea, fungi and other eukaryotes (Baggs, 2011). Part of the denitrifying bacteria and archaea are missing the genes encoding the enzymes involved in the reduction of nitric oxide (NO) and N_2_O to N_2_, which can lead to incomplete pathways and N_2_O release (Stein & Klotz, 2016). Denitrification enzymes are inhibited by O_2_, particularly N_2_O reductase, which catalyzes the reduction of N_2_O to N_2_. Thus, under suboxic conditions, N_2_O may be the end product of denitrification (Knowles, 1982). Apart from O_2_ conditions, several other environmental factors control N_2_O production from denitrification, specifically the N_2_O yield (N_2_O/(N_2_O+N_2_)), including water content, NO${}_{3}^{-}$ availability, C quality and availability and C:NO${}_{3}^{-}$ (Quick et al., 2019).

Nitrification and nitrifier denitrification occur under different environmental conditions and both oxidize ammonia. Nitrification is the oxidation of ammonia (NH_3_) or ammonium (NH${}_{4}^{+}$) to NO${}_{2}^{-}$ by ammonia oxidizers (cohort I; primary nitrifiers) and to NO${}_{3}^{-}$ by nitrite oxidizers (cohort II; secondary nitrifiers). Ammonia/um can be directly oxidized to nitrate by complete ammonia oxidizers (comammox, cohort III). Cohorts II and III only include chemolithotrophic microbes (Stein & Klotz, 2016). Under certain conditions, ammonia oxidizers can significantly contribute to N_2_O emissions by two reactions along this pathway, hydroxylamine oxidation (biotic and abiotic) and chemodenitrification (abiotic). The main factors influencing hydroxylamine oxidation are aerobic conditions and NH_3_ availability, whilst chemodenitrification is limited by NO${}_{2}^{-}$ availability and may occur under fluctuating redox conditions (Quick et al., 2019). Nitrifier denitrification, strictly carried out by ammonia oxidizers, converts NH_3_ to N_2_ gas. It is supported by different O_2_ conditions, having both oxidation and reduction steps. The first steps are oxidative (ammonia is oxidized to NO${}_{2}^{-}$) and the final steps are reductive (NO${}_{2}^{-}$ is sequentially reduced to NO, N_2_O and N_2_). Factors influencing N_2_O production from nitrifier denitrification include O_2_ conditions, NH${}_{4}^{+}$ and C availability (Quick et al., 2019). It differs from nitrification and coupled nitrification-denitrification as there is no NO${}_{3}^{-}$ involved.

DNRA is performed by both bacteria and fungi, using C as an electron donor. During nitrate ammonification, nitrate is reduced to NO${}_{2}^{-}$ and NH${}_{4}^{+}$, and N_2_O is produced as a by-product during the NO${}_{2}^{-}$ reduction stage. Reducing conditions are an important factor controlling this process, which is mostly anaerobic but can also occur under relatively oxic conditions, being less sensitive to O_2_ than denitrifiers (Giles et al., 2012). The C:NO${}_{3}^{-}$ ratio is also considered an important controlling factor in the process (Quick et al., 2019).

Although this synthesis focuses on freshwater sediments as net sources of N_2_O, it is important to acknowledge that N_2_O uptake also occurs in aquatic systems, which may act as N_2_O sinks (Syakila, Kroeze & Slomp, 2010; Soued, Giorgio & Maranger, 2015). N_2_O consumption has been associated with low mineral content, large moisture contents and decreased gas diffusivity and various processes can contribute to N_2_O consumption, such as denitrification and nitrifier nitrification, however N_2_O consumption is often masked by higher rates of N_2_O production (Chapuis-Lardy et al., 2007; Kroeze, Bouwman & Slomp, 2007).

## Effects of dry-wet cycles on N_**2**_O emissions in freshwater sediments

Lotic and lentic systems can experience intermittent wet and dry conditions of varying duration (seasonal, ephemeral, and episodic) and intensity (partial or complete drying). As a notable fraction of watercourses is temporary (Datry, Larned & Tockner, 2014), and even small lentic systems can have a large contribution to greenhouse gas (GHG) emissions (Holgerson & Raymond, 2006), dry-wet cycles in these systems potentially play a major role on N_2_O emissions. Moreover, N_2_O pulses exceeding CO_2_ pulses after rewetting emphasizes the need to further explore the effects of drying and wetting cycles on N_2_O production (Beare, Gregorich & St-Georges, 2009).

Water content and water level fluctuations in sediments during dry-wet cycles exert a strong influence on key factors controlling C and nitrogen (N) cycling in temporary systems, namely microbial community structure and functioning, and organic matter (OM) quality and availability (Reverey et al., 2016). At the microsite scale, moisture and water saturation are particularly important in controlling the processes leading to N_2_O production by creating oxic-anoxic conditions along the sediment strata. Biogeochemical processes such as nitrification and denitrification are therefore affected by dry-wet cycles, potentially impacting N_2_O production and emission. Microbial activity is strongly coupled with water-filled pore space (WFPS), a measure of moisture’s influence on biogeochemical processes which normalizes the effect of texture (Aulakh, Doran & Mosier, 1992). Maximum N_2_O flux rates are usually reported to occur between 60 and 70% WFPS, but maximum rates have been observed over a wide range of %WFPS (Castellano et al., 2012). Differences in the sediment-water retention capacity will lead to spatial and temporal differences in sediment drying.

### Effects of drying on N_2_O emissions in freshwater sediments

**Figure 2:**
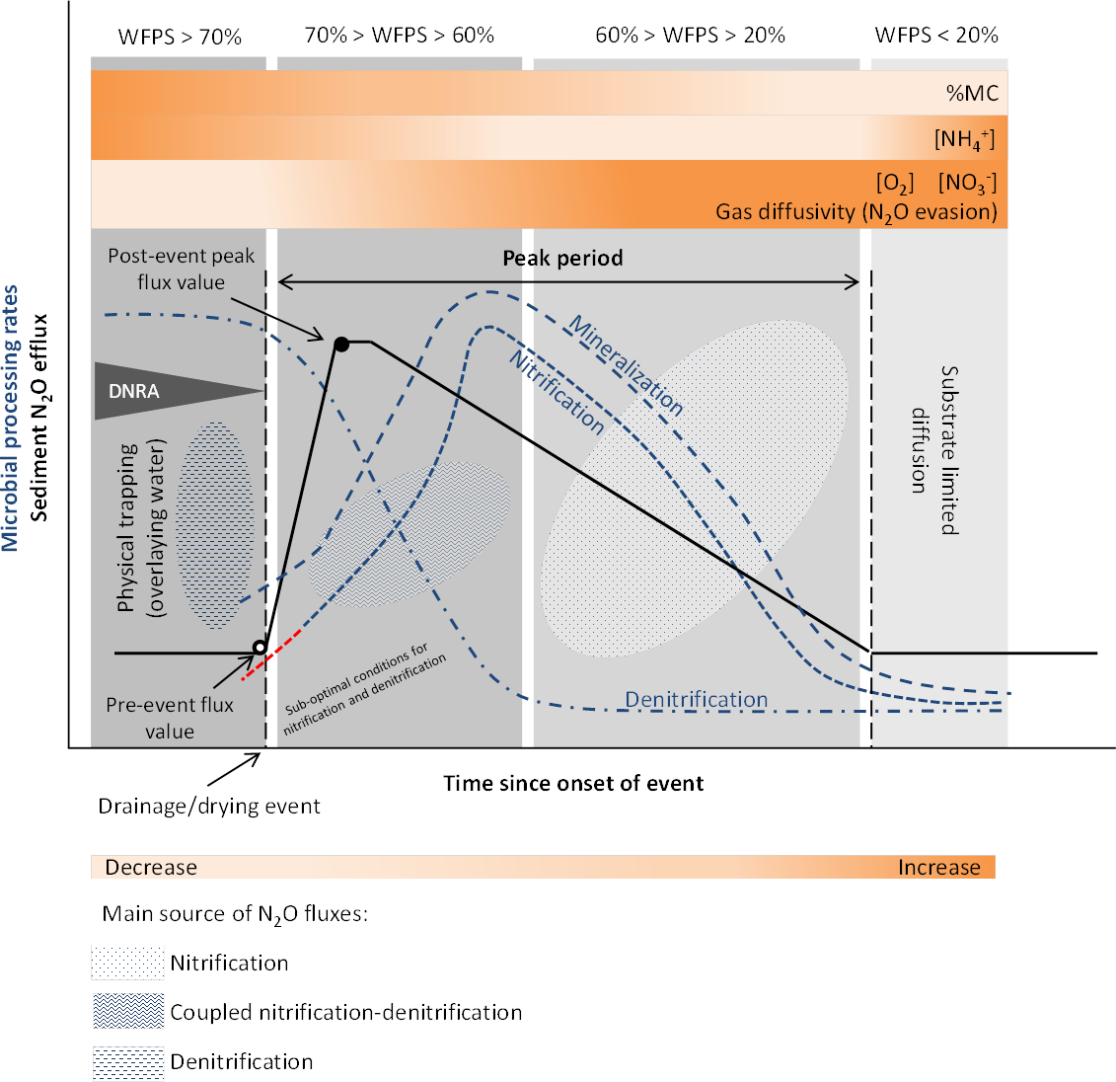
Conceptual diagram of N processing rates and N_2_O efflux during sediment desiccation. Dynamics of pore water (reference %WFPS thresholds, e.g., Bateman & Baggs, 2005) affect microbial processing rates and physical mechanisms leading to N_2_O production, trapping and evasion. Different N_2_O producing processes may occur simultaneously at varying %WFPS, including nitrifier denitrification (not represented on the diagram). A lag phase (red –) in the reactivation of nitrification (adaptation) is observed after aeration. Increased N_2_O production and emission occur when conditions are suboptimal for nitrification and denitrification (coupled nitrification-denitrification). N_2_O emissions are highest at an optimum MC/WFPS where gas and substrate diffusion are suitable, and decline at drier or wetter conditions. Wetter sediment conditions limit gas diffusivity (physical trapping) while drier conditions facilitate gas diffusion (N_2_O evasion) but limit substrate diffusion to reaction sites. NO${}_{3}^{-}$ accumulates in the sediment as denitrification rates decrease faster than nitrification rates. NH${}_{4}^{+}$ decreases as a result of nitrification. NH${}_{4}^{+}$ can accumulate in the sediment with ongoing desiccation if mineralization exceeds nitrificaton rates, and by microbial release from cell lysis. Simplified gas flux dynamics (adapted from Kim et al., 2012, CC BY 3.0 SA).

Both physical mechanisms and microbial processes have been suggested to contribute to the initial N_2_O pulse during sediment desiccation. The increase in gas diffusivity (i.e., the decrease in mass transfer resistance) and the lack of physical trapping, related to water content and oxygen fluctuations, determine the magnitude and temporal development of N_2_O outgassing from the sediments (Reverey et al., 2016). Higher N_2_O emissions are also attributed to periods of enhanced microbial activity due to coupled nitrification-denitrification occurring at the boundary of oxic-anoxic environments (Baldwin & Mitchell, 2000) (Fig. 2).

Drying provides suitable aerobic conditions for nitrification to occur and in turn, the NO${}_{3}^{-}$ and NO${}_{2}^{-}$ produced are reduced by denitrification in anoxic microsites, harbored within the oxic parts of the sediment. Substrates for N_2_O production are also made available from the release of intracellular solutes from microbial cell lysis, and from mineralization of OM, including previously protected organic matter from aggregate disruption (shrinking), due to sediment desiccation (Baldwin & Mitchell, 2000; Borken & Matzner, 2009).

In general, studies in both lotic and lentic systems suggest that initial sediment desiccation stimulates N processing rates such as N mineralization and nitrification. A MC/WPFS threshold exists after water drainage and/or the onset of sediment drying when denitrification is also enhanced and coupled to nitrification (Fig. 2). This was observed in sediments from intermittent rivers and streams, where N mineralization and nitrification were found to be stimulated during initial sediment desiccation, while denitrification significantly decreased as %WFPS declined (Gómez et al., 2012). Between the onset of drying and the increase in net nitrification rates, nitrification and denitrification occurred simultaneously (coupled nitrification-denitrification), as evidenced by the NO${}_{3}^{-}$–N concentrations and enhanced denitrification rates detected during this time (Gómez et al., 2012). NO${}_{3}^{-}$–N tends to accumulate in the sediment with ongoing drought, while NH${}_{4}^{+}$–N tends to decrease (Fig. 2). During the first days or weeks of sediment desiccation, the increase in NO${}_{3}^{-}$–N content and loss of NH${}_{4}^{+}$–N most likely results from continuous nitrification throughout the dry period (Gómez et al., 2012; Arce et al., 2014; Merbt et al., 2016; Arce et al., 2018; Table 1). Furthermore, and similarly to highlighted observations for soils (Borken & Matzner, 2009), the duration of the desiccation period also controls the N concentration in the sediments (Gómez et al., 2012), with consequences for the N_2_O/(N_2_O + N_2_) ratios, since denitrifiers prefer to transfer electrons to NO${}_{3}^{-}$ rather than N_2_O.

Enhanced N_2_O fluxes have been observed in different freshwater systems during initial sediment desiccation (Fig. 2). In an intermittent stream, Arce et al. (2018) found an N_2_O peak average value after 11 days of sediment drying, after which N_2_O fluxes decreased. This pulse in emissions was attributed to physical N_2_O evasion favored by increased gas diffusivity (Fig. 2). The sources of N_2_O fluxes were assumed to be denitrification under wet to saturated conditions, and nitrification as O_2_ concentration increased. Dry sediments can be active spots for N_2_O emissions even at low moisture contents (Pinto et al., 2020), underlining the importance of including dry phases in the N emission estimates.

In a seasonal floodplain lake, higher N_2_O emissions were measured during the transition from flooded to dry sediment conditions (Koschorreck, 2005; Table 1). The majority of N_2_O loss (94% of the total N_2_O emission) occurred during an initial drying period of high N processing activity (59% of the total denitrification). After an initial lag phase, most likely limited by NO${}_{3}^{-}$ supply via nitrification, denitrification activity and N_2_O flux peaked. During an intermediate state between flooding and sediment drying, a patchy distribution of oxic and anoxic microsites was observed. This high spatial heterogeneity in redox conditions increases the area of the oxic-anoxic boundary where coupled nitrification-denitrification can occur, explaining the high denitrification activity detected during this period. The denitrification rates during desiccation exceeded those during re-flooding. A previous study in the same system detected higher N_2_O and denitrification rates in exposed sediments, and with greater distance to the waterline (Kern et al., 1996; Table 1). Namely, O_2_ availability was the main factor controlling N_2_/N_2_O ratios. The coupling of nitrification and denitrification was also suggested, due to the lack of NO${}_{3}^{-}$ accumulation in the sediments.

**Table 1:** Summary of nitrogen dynamics during dry-wet cycles (cited literature): Settings, N-cycling pathways, nitrogen species and N_2_O emissions.

Ref	Settings	N dynamics			Observations
		N processes	Mineral N	N_2_O emissions	
Lentic systems					
Kern et al. (1996)	Seasonal floodplain lake (Brazil) Aquatic/terrestrial interface: exposed sediments (*in situ* incubation) vs. flooded sediments (laboratory incubation) N_2_O^(1)^ (*in situ* field measurements, glass domes inserted into the sediment; laboratory incubation measurements, flasks)	Denitrification: - Higher rates in exposed sediments (increase with distance to the water line) - Flooded sediments: detected only during low water	NO${}_{3}^{-}$ No accumulation in exposed sediments (pore water) NH${}_{4}^{+}$ High in exposed sediments (pore water)	Exposed sediments (increase with distance to the water line; average rate; MC >50%/waterlogged): - 1 m from the water line: 256.2 µg N m^−2^ h^−1^ - 5 m from the water line: 396.2 µg N m^−2^ h^−1^ Flooded sediments: not detected	Denitrification rates in flooded sediments 1 –2 orders of magnitude smaller than in temperate regions. Nitrate removal of exposed sediments higher than in undisturbed wetland soils on temperate regions. Strong coupling of nitrification and denitrification in exposed sediments. High N release during transition from the aquatic to the terrestrial ecotope (high impact on gaseous N turnover)
Merbach et al. (2002)	Kettle holes (NE, Germany) Sampling along a depositional sequence with different hydrological conditions: periodically flooded (terrestrial zone), drying-out periodically (lower bank), and drying-out episodically (shallow water zone) N_2_O (*in situ* field measurements, static flux chambers)	_	_	Decreased with higher water level and increased with lowering of the water table Generally higher in the terrestrial zone	Environmental factors (water level) superimposed relations between available N compounds and trace gas emissions
Huttunen et al. (2003)	Boreal lake (Finland) Sampling sites: temporarily flooded littoral zone (A), upper littoral infralittoral (B), continuously flooded littoral zone (C) Year 1997 (extremely dry): water table <0 (site A) and <0 (site B and C); Year 1998 (extremely wet): water table >0 (all sites) N_2_O^(2)^ (*in situ* field measurements, static flux chambers)	_	_	Site A and B (1997): N_2_O emissions in the driest part of the littoral zone ranged from 11 ± 7 to 22 ± 7 µg N m^−2^ h^−1^; Peaks = 140 µg N m^−2^ h^−1^ (site A) and 59 µg N m^−2^ h^−1^ (site B) Site C: N_2_O flux near zero Higher emissions during the dry vs. wet summer 15 (32) ± 8 µg N m^−2^ h^−1^ and6 (15) ± 2 µg N m^−2^ h^−1^, respectively (mean, median, standard deviation)	The littoral zone occupied 26% of the lake area but was estimated to account for most of the N_2_O emissions from the lake.
Koschorreck (2005)	Seasonal floodplain lake (Brazil) Sampling: shallow part which dries out for ≈ 3 months Dry period: 55 d N_2_O (*in situ* field measurements, glass domes inserted into the sediment)	Denitrification: - Lag phase [0 –2 d] - Increase [2 –5* d] - Peak: 574 µg N m^−2^h^−1^) DEA (surface sediments): - Increase [0 –5* d] - Peak: 406 µg N h^−1^ g DW^−1^	NO${}_{3}^{-}$ Low content, little change with depth and time NH${}_{4}^{+}$ - Decrease [0 –40 d] - Constant low [40 –55 d]	Lag phase [0 –2 d] Increase [2 –5* d]; Peak: ≈ 245 µg N h^−1^ m^−2^	Highest inorganic N loss occurred during the first 10 days of the drying period. Inorganic N loss was higher in the deeper (1 –5 cm) vs. surface layer (0 –1 cm). Cell numbers of nitrate reducers increased in the deeper layer. Coupled nitrification-denitrification was the main mechanism of N removal in the sediments, with peak activity shortly after drying.
Hernandez & Mitsch (2006)	Artificial marsh (Ohio, USA) Sampling along a transect with different hydrological conditions: dry (edge zone), alternate dry-wet (high marsh zone) and permanently flooded (low marsh zone) sediments N_2_O^(2)^ (*in situ* field measurements, static flux chambers)	_	NO${}_{3}^{-}$ Low marsh <open water <high marsh <edge zones NH${}_{4}^{+}$ Low marsh >open water >high marsh >edge zones Total N Similar between low marsh, high marsh and open water and significantly higher than in the edge zone	Edge zone (dry): - Before flood pulse 4.1 ± 1.8 µg N m^−2^ h^−1^(significantly lower) - During flood pulse: 11.3 ± 3.1 µg N m^−2^ h^−1^ - After the flood pulse: 7.3 ± 3.3 µg N m^−2^ h^−1^ High marsh (dry-wet): - Before flood pulse: 2.4 ± 6.5 µg N m^−2^ h^−1^ [WL = -0.22 m] - During flood pulse: 6.9 ± 2.2 µg-N m^−2^ h^−1^ [WL = 0.16 m] - After flood pulse: 25.9 ± 13.8 µg-N m^−2^ h^−1^ (significantly higher) [WL = -0.09 cm, just below the surface] Low marsh zone (permanently flooded): low flux rates, regardless of the flood pulse condition	Surface flooding was infrequent at the edge zone (dry) but ground-water levels changes affected emissions. Low and high marsh: the total nitrogen content was significantly lower in deeper (8–16 cm) vs. top (0–8 cm) layer.
Fromin et al. (2010)	Mediterranean seasonal pond (SE France) Sampling at different periods of drying and rewetting: drainage, dry, rainfall (after several weeks of drying) Dry period: 8 w Wet period: rain event; 48 h	NEA (dry period): - Null or very low (Surface sediments); 0 –0.4 µg nitrified-N g^−1^ DW h^−1^ - Increase (Subsurface sediments); 0.6 –1.1 µg nitrified-N g^−1^ DW h^−1^ NEA (rain event): - Increase (Surface sediments) - ns (Subsurface sediments) DEA (dry period): - Low in flooded sediments (0.2 –0.6 µg N_2_O-N g^−1^ DW h^−1^) - Increase [0 –5 w]; 0.8 –2.6 µg N_2_O-N g^−1^ DW h^−1^(Surface sediments) - Decrease [6 –8 w]; mean value: 1 µg N_2_O-N g^−1^ DW h^−1^ (Surface sediments) DEA (rain event): - Increase (Surface sediments) - ns (Subsurface sediments)	Total N Increase during drying period	_	Proportion of N potentially denitrified as N_2_O was positively correlated to the duration of dry period. Rain triggered surface DEA yet values were not significantly higher than the maximum rates observed during mid-drought period. DEA rates after the rain event were higher in pond margins sites (hot spots; McClain et al., 2003). Subsurface sediments (2-10cm) were less affected by MC variations than surface (0-2cm) sediments. Bacterial density was significantly lower in the surface vs. subsurface layer.
Akatsuka & Mitamura (2011)	Lake (Japan) Sampling sites: littoral wetland sediments in the flooded and exposed region; sediments in the exposed region selected at various distances from the water line (varying degrees of dryness) Sediment cores from the flooded region were exposed to the atmosphere (10 days; drying treatment) and sediment cores from the exposed region were flooded (2 weeks; wetting treatment) N_2_O measured from the denitrification assay under no addition of acetylene (units of volume)	Potential denitrification rate (with nitrate addition) higher than the denitrification rate (no nitrate addition) Potential denitrification rates and denitrification rates higher in the exposed vs. flooded region	NO${}_{3}^{-}$ - Undetectable in the flooded region - Concentration increased with the degree of sediment dryness in the exposed region NH${}_{4}^{+}$ Concentration increased with wetting and decreased with drying	With nitrate application: - Before and after wetting treatment: 0.74 and 0.32 µg N cm^−3^h^−1^, respectively - Before and after drying treatment: 0.031 and 0.38 µg N cm^−3^h^−1^, respectively Negligible under no nitrate application	Nitrous oxide emission/denitrification ratios decreased in the wetting treatment (from 55 to 23%) and increased in the drying treatment (from 18 to 70%)
Wang et al. (2012)	Lake (N China) Sampling along a littoral gradient: deep sediment (A), near-transition sediment (B), transition site (C), near-transition land (D) and land soil (E) N_2_O measured from the denitrification assay under no addition of acetylene	Potential denitrification rate in sediments (A and B) 25 times higher than soils (D and E)	NO${}_{3}^{-}$ Content significantly lower in sediments than soils NH${}_{4}^{+}$ Deep sediment and near-transition sediment: 150 times higher content than soils	Potential N_2_O production rate in sediments 3.5 times higher than soils	The N_2_O/(N_2_O+N_2_) ratio of sediments was seven times lower than in land soils (higher proportion of N_2_O transformed into N_2_ in sediments)
Jin et al. (2016)	Hydroelectric reservoir (S Korea) Sampling sites with different hydrological conditions: flooded sediments, recently expose (moist) sediments, drier sediments N_2_O (laboratory incubation)	_	_	Significantly higher in recently exposed sediments Peak (mean): ≈ 11.46 µg N m^−2^h^−1^ Flooded sediments (mean): <≈ 0.63 µg N m^−2^d^−1^	N_2_O production was suggested to derive from both aerobic and anaerobic processes.
Reverey et al. (2018)	Kettle holes (NE Germany) Sediment cores Sampling sites with different hydrological history: predominantly inundated (zone A), intermediate/occasionally dry (zone B), sediments frequently exposed to dry-wet cycles (Zone C); (sampling during sediment exposure) Exposed vs. inundated experiments	Nitrification NH${}_{4}^{+}$ depleted in zones B and C (exposed phase), likely nitrified to NO${}_{3}^{-}$ DEA >DNRA in all zones DNRA Higher potential in sediments less exposed to drought (NH${}_{4}^{+}$ accumulation coupled to NO_3_^−^depletion during exposed phase)	NO${}_{3}^{-}$ - Exposed conditions: lowest concentration in zone A - Inundated conditions: decreased significantly after rewetting in zones B and C; similar concentrations between zones NH${}_{4}^{+}$ - Exposed conditions: highest concentration in zone A - Inundated conditions: highest concentration in zone A and lowest in zone C; increased significantly in all three zones after rewetting	_	Water content of the sediment did not drop below 80% (zones A and B) and 60% (zone C).
Shi et al. (2020)	Hydroelectric reservoir (China) 10 sampling sites along a transect after the water level receded (0.5, 1.5, 3.5, 6.5, 10.5, 15.5, 20.5, 25.5, 30.5, and 35.5 m) Sediment core system (*in situ*) Wetting-drying cycles with different periods or water level rising and dropping N_2_O (*in situ* field measurements after the water level receded, static flux chambers)	Denitrification: - Strong positive relationship with the period of water level rising-falling cycle; - Increasing potential from the water edge to the center of the transect (0.5 –10.5 m), followed by a reduction for longer distances (15.5 –35.5 m)	NO${}_{3}^{-}$ Below detection limit (0.5 to 25.5 m)	Similar pattern to the observed for denitrification potential rate: - Increase from the water edge to the center of the transect (0.5 –10.5 m): 51 to 62 µg N m^−2^ h^−1^ - Decrease to 0.27 µg N m^−2^ h^−1^ (35.5 m)	Denitrification more active in the surface sediment than in the subsurface sediment.
Lotic systems					
Austin and Strauss (2011)	Experimental stream (outdoor; NE Kansas) Dry period: 1, 3, 7, 14, 21, 28 d Wet period: 1, 3, 7, 14, 21, 28 d	Nitrification: - Dry period: decrease (after 1 d) - Wet period: recovery in sediment dried for 1, 3, 7 (after 1 d rewetted) and 21 d (after 3 d rewetted); sediments dried 14 and 28 d increased but failed to recover; rates declined below control rates after recovery DEA - Dry period: decrease (after 3 d) - Wet period: recovery in sediment dried for 3, 7 and 21 d, after 7 d rewetted; sediment dried for 1 d (ns); sediment dried for 14 and 28 d failed to recover	NH${}_{4}^{+}$ - Dry period: increase - Wet period: decrease	_	Lag between rewetting and recovery of process rates. Denitrifiers appear to be more diverse and/or drought tolerant than nitrifiers. Water availability appears to regulate N cycling during drying, and O_2_ availability was more important during rewetting.
Gómez et al. (2012)	Mediterranean intermittent stream (S Spain) Microcosms (outdoor) Dry period: 18 d	Nitrification: - Lag phase [0 –4 d] - Increase [4 –8* d] - Decrease [8 –18 d] Denitrification: - Peak (24 h) - Inhibited [1 –18 d] Mineralization stimulated during the first days	NO${}_{3}^{-}$ - Increase [0 –10* d] - Decrease [10 –18 d] NH${}_{4}^{+}$ Decrease [4 –18 d]	_	Denitrification pattern differs from (Kern et al., 1996; Koschorreck, 2005) which reported an increase in denitrification with drying. This was attributed to differences in MC (8% vs. >50% and 25–50%, respectively) and better sediment aeration.
Shrestha et al. (2012)	Flash flow river regime (River Thur, NE Switzerland) Sampling sites in different functional process zones, FPZs (flooding frequency, yr^−1^): frequently flooded gravel bars (>10), bank (4-6), forests (1-2) and embankment (1-2) N_2_O (*in situ* field measurements, static flux chambers)	GN >GM DEA >GN	NO${}_{3}^{-}$ - Slower turnover rates (compared to NH${}_{4}^{+}$) - Higher in gravel bar and forest NH${}_{4}^{+}$ Fast turnover rate	The N_2_O efflux generally increased as a result of flood disturbance	N_2_O efflux did not correlate with denitrification, suggesting that: (i) other processes also contributed to N_2_O emissions, namely nitrification, or (ii) production in deeper layers. N turnover governed by mineralization. NO${}_{3}^{-}$ quickly denitrified during water saturation, and during drying phases in anoxic microsites. Under predominant aerobic conditions, NH${}_{4}^{+}$ not consumed or immobilized is quickly nitrified, except when conditions are too dry. The N_2_O efflux generally increased as a result of flood disturbance.
Shrestha et al. (2014)	Flash flow river regime (River Thur, NE Switzerland) Sampling sites in different functional process zones, FPZs (flooding frequency, yr^−1^): frequently flooded gravel bar (>10), bank (4-6), forest (1–2)	GN stimulated during the drying phase (gravel bar) DEA: - Increased after flood events - Temporary drop a few days after flood (gravel bar) GM: - Strongly increased immediately after flood events (gravel bar) - Temporary dropped during the drying phase after the flood (possible leaching of available organic N)	NO${}_{3}^{-}$ Marked temporary drop in gravel bar (negative correlation with DEA) NH${}_{4}^{+}$ Increase after flood events	Hot moments of N transformations may lead to temporary high N_2_O emissions	Stronger reaction of N pools and transformation rates to flooding, in the gravel bar. High N turnover by coupled nitrification–denitrification during the drying phase after a major flood.
Arce et al. (2014)	Mediterranean temporary stream (intermittent reach) (SE Spain) Sampling during dry and wet periods	Nitrification: - Higher in rewet sediments (incubation with stream water) than during wet period - Quick recovery to pre-dry levels or higher upon rewetting (12h) Denitrification: - Similar between dry and wet period - Quick recovery to pre-dry levels or higher upon rewetting (5h)	NO${}_{3}^{-}$ - Increase (dry period) - Decrease (wet period) NH${}_{4}^{+}$ ns between wet and dry period	_	Rapid recovery (hours) of processing rates after drying period under natural conditions (4 months). Austin and Strauss (2011) report a variable lag (∼30 d) between rewetting and recovery of processing rates, depending on the pre-drought period (experimental study) in a temperate stream. The lack of a negative effect of duration of the sediment desiccation may be related to field conditions, e.g., occurrence of small water/rainfall pulses (field vs. experimental study).
Gallo et al. (2014)	Semi-arid urban ephemeral waterway (Tucson, Arizona) Wet period: artificial rainfall event (10 mm; after extensive dry period of 3 –4 months) N_2_O (*in situ* field measurements, static flux chambers)	_	_	Pre-wetting: 1.5 ± 0.6 µg N m^−2^ h^−1^ Post-wetting: 207.4 ± 76.3 µg N m^−2^ h^−1^ Instantaneous: 458.6 ± 237.7 µg N m^−2^ h^−1^	N_2_O emissions following wetting were among the highest ever published.
Arce, Sánchez-Montoya & Gómez (2015)	Mediterranean temporary stream (intermittent reach) (SE Spain) Microcosms (outdoor) Wet period: 7 d (after 3 months sediment desiccation)	Denitrification: - Peak (24 h) - Decrease [24 h–7 d] DNRA (low Eh)	NO${}_{3}^{-}$ - Increase [0 –1 h] - Decrease [1 h –7 d] despite low denitrification NH${}_{4}^{+}$ Increase (DNRA)	_	Rapid recovery (hours) of denitrification rates after drying period under controlled conditions (3 months).
Merbt et al. (2016)	Mediterranean intermittent stream (NE Spain) Sampling sites with different hydrological regimes, including short-term dry (5 d) and long-term dry (30 d)	Increase (AO)	NO${}_{3}^{-}$ Increase NH${}_{4}^{+}$ Increase (cell lysis)	_	The increase in AO activity and nitrate content with the degree of stream drying was more evident in surface sediments.
Arce et al. (2018)	Temperate intermittent river (Germany) Microcosms (laboratory) Dry period: 9 w Wet period: rainfall pulses (after 6 and 9 w drying); modest (4 mm) and intense (21 mm) N_2_O (laboratory, gas-tight microcosms)	Nitrification: - Decrease (AO; surface sediments) - Stable (AO; deep sediments) COMAMMOX (uncertain)	NO${}_{3}^{-}$ Increase with drying NH${}_{4}^{+}$ - Decrease [0 –3 w ] - Constant low [3 –9 w]	Increase (-3 –11* d); Peak: 182.5 µg N m^−2^h ^−1^ Decrease (2 –9 w); range: 0 –5.6 µg N m^−2^h ^−1^ Immediately after drainage (0 d): ≈ 5 µg N m^−2^h^−1^ (≈ 0 µg N m^−2^h^−1^ during the preceding flooded conditions) Immediately after rainfall pulses (0 h) (4 mm and 9 mm rainfall, respectively): - 6 weeks desiccation period: ≈ 6 and 19 µg N m^−2^h^−1^ (≈ 6 and 17 µg N m^−2^h^−1^ during the preceding dry conditions, respectively) - 9 weeks desiccation period: ≈ 17 and 0 µg N m^−2^ h^−1^ (≈ 0 µg N m^−2^h^−1^ during the preceding dry conditions)	Based on MC conditions (wet-saturated), denitrification was assumed to be the dominant source of N_2_O emissions during the first 11 days. An increase in AO in the sediments before the first sampling time (3 weeks) was probably overlooked. Rewetting had a negative impact on N_2_O emissions as its magnitude increased (physical trapping). NO${}_{3}^{-}$ increase was not as high in deeper (3 –15 cm) vs. surface (0 –3 cm) sediments. NH${}_{4}^{+}$ decrease more pronounced in deep vs. surface layers (higher initial NH${}_{4}^{+}$ concentrations in deeper sediments).
Pinto et al. (2020)	Temperate river (Lower Austria) Sampling sites along an hydrological gradient (parafluvial zone): frequently flooded sediments, rarely flooded sediments, and non-flooded floodplain soil Sampling periods defined as a function of water level fluctuations at the parafluvial zone: intermittent, desiccation and post flood period N_2_O (*in situ* field measurements, static flux chambers)	_	NO${}_{3}^{-}$ - Frequently flooded sediments: Intermittent >post flood >desiccation period - Rarely flooded sediments: similar concentrations between periods NH${}_{4}^{+}$ - Highest concentrations in frequently flooded sediments, except during the desiccation period (enhanced mineralization) - Strong increase after the flood in rarely flooded sediments	Intermittent period: 37 ± 24 µg N m^−2^ h^−1^ Desiccation period: 13 ± 16 µg N m^−2^ h^−1^ Post flood period: 19 ± 27 µg N m^−2^ h^−1^ Frequently flooded sediments: 32 ± 25 µg N m^−2^ h^−1^ Rarely flooded sediments: 23 ± 19 µg N m^−2^ h^−1^ Non-flooded soil: 27 ± 33 µg N m^−2^ h^−1^ (mean ± SD)	Tight link between C and N cycles, presumably originating from the quality of the DOM pool.

**Notes.**

(*) indicates peak timing; Periods in hours (h), days (d), weeks (w); ns: not significant; AO – ammonia oxidation; DEA – potential denitrification; NEA – potential nitrification; GN – gross nitrification; GM – gross mineralization; DW – dry weight. N2O emissions from headspace measurements

Similar observations were made in a seasonal pond (Fromin et al., 2010; Table 1). There, potential denitrification was strongly stimulated during early desiccation of the sediment, while potential nitrification was stimulated during the late desiccation period. The potential for N_2_O emissions was higher at sites with a longer dry period, pointing to different response magnitudes of the microbial processes depending on the length of the dry period. Both nitrification (at depth) and denitrification enzyme activities remained very high in sediment after 1 month of drought, and communities were able to maintain their enzymatic pool even under severe environmental conditions. Short-term variations in sediment water content strongly affected microbial functioning, but not the metabolic structure of the community.

During an extreme drought event in a hydroelectric reservoir, N_2_O production potentials were measured along one transect, from flooded sediments to drier sediments at the water margin (Jin et al., 2016; Table 1). N_2_O production was significantly higher in recently exposed sediments than in flooded or dry sediments, related to a temporary boost in OM processing in the drying sediment (Fig. 2). The increase in N_2_O production as a consequence of enhanced O_2_ availability during drying suggested that N_2_O also derived from aerobic processes such as nitrification.

### Effects of rainfall events on N_2_O emissions in freshwater sediments

**Figure 3:**
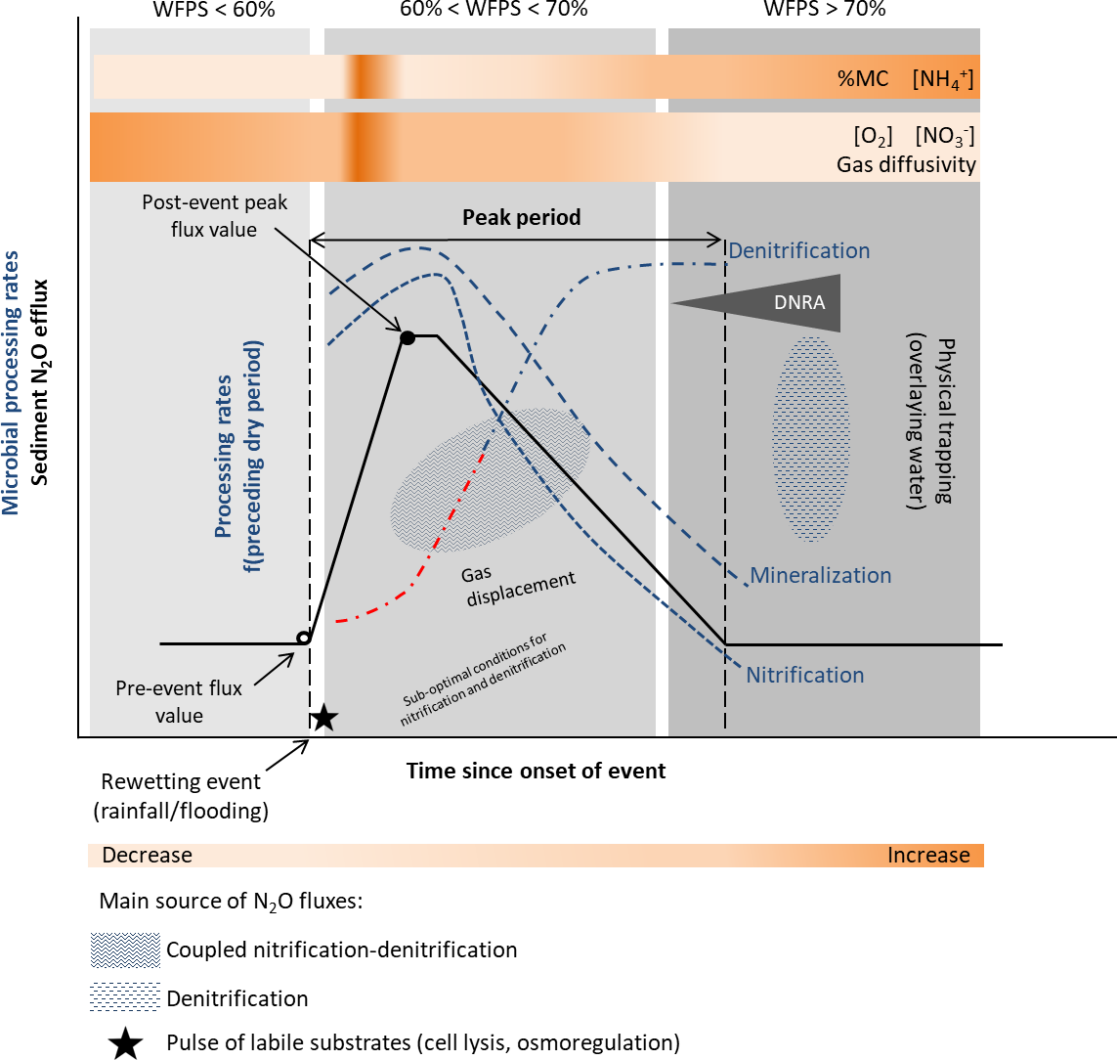
Conceptual diagram of N processing rates and N_2_O efflux during rewetting. Dynamics of pore water (reference %WFPS thresholds, e.g., Bateman & Baggs, 2005) affect microbial processing rates and physical mechanisms leading to N_2_O production, displacement and trapping. N processing rates before the rewetting pulse depend on the duration and intensity of the preceding dry period [f(preceding dry period)]. A lag phase (red −⋅) in the reactivation of denitrification (recovery) is observed after the dry period, and depends on the duration and intensity of the preceding dry period. Different N_2_O producing processes may occur simultaneously at varying %WFPS. Increased N_2_O production and emission occur when conditions are suboptimal for nitrification and denitrification (coupled nitrification-denitrification). Upon rewetting, labile substrates are made available for N_2_O production due to cell lysis from excessive influx of water into the microbial cells, and as microbes secrete cytoplasmic solutes for osmotic regulation (nutrient pulse). Decomposition of labile OM and N processing is enhanced. Physical gas displacement by water contributes to the N_2_O pulse, as changes in the water pressure can remove gas pockets compensated by a liquid flow into the sediment. N_2_O entrapment occurs with prolonged saturation/inundation (decrease in gas diffusivity). NO${}_{3}^{-}$ decreases and NH${}_{4}^{+}$ increases in the sediment as denitrification rates increase and nitrification rates decrease, respectively. When present, DNRA can remove NO${}_{3}^{-}$ and provide NH${}_{4}^{+}$. Simplified gas flux dynamics (adapted from Kim et al., 2012, CC BY 3.0 SA).

Recent studies recognize that as in soils, the Birch effect, a pulse in decomposition, mineralization and release of inorganic nitrogen and CO_2_, is also observed in sediments (Birch, 1958; Wilson & Baldwin, 2008; Arce et al., 2019; Marcé et al., 2019). In addition, similar responses to a wetting pulse have been observed for nitrification and denitrification processes (Arce et al., 2014; Arce, Sánchez-Montoya & Gómez, 2015), with enhanced N and C mineralization increasing nitrogen turnover rates and N_2_O emissions (Fromin et al., 2010), following a desiccation period. The main reasons leading to increased fluxes are enhanced microbial activities (including nitrification and denitrification), induced by a pulse of labile substrates (cell lyses, osmoregulation), and physical mechanisms such as gas displacement (Baldwin & Mitchell, 2000; Fierer & Schimel, 2003; Borken & Matzner, 2009; Congreves et al., 2018) (Fig. 3). The release of previously protected organic matter due to aggregate slaking may also contribute to the nutrient flush upon rewetting (Denef et al., 2001; Shrestha et al., 2012).

Nitrification and denitrification response to rewetting following dry periods of varying duration have yielded contrasting results. In an experimental stream, nitrification and denitrification rates recovered to levels equal or greater than those in the wet controls within 1 day of being rewetted in sediments dried less than 7 days, but failed to fully recover in sediments dried more than 7 days (Austin and Strauss, 2011; Table 1). On the other hand, the negative effect of desiccation duration was not detected upon rewetting of sediments from temporary streams (Arce et al., 2014; Arce, Sánchez-Montoya & Gómez, 2015; Table 1). A rapid recovery of N processing rates (within hours) to pre-drought levels or higher was observed, particularly for denitrification (Arce et al., 2014). After 1 h of sediment rewetting, there was a significant stimulation of denitrification rates and a peak after 24 h was detected (Arce, Sánchez-Montoya & Gómez, 2015). These results differed from those of temperate streams, which reported higher lag time between rewetting and recovery of denitrification rates to the pre-dry levels (Zaman & Chang, 2004). The lack of a clear microbial response to the rewetting stress suggests a lag phase between physiological adjustments or resilience of microbial communities to changing environmental conditions. A high export of NO${}_{3}^{-}$ (accumulated during sediment drying) was also observed during the first flushing events (Arce et al., 2014; Arce, Sánchez-Montoya & Gómez, 2015). Previous studies have highlighted the influence of the timing and duration of the drying-rewetting events on N availability and process dynamics (Gómez et al., 2012).

In ephemeral stream channels, N_2_O emissions were significantly higher after a rainfall pulse following an extensive period of dry conditions (Gallo et al., 2014; Table 1). During an initial stage, the extremely high instantaneous fluxes were attributed to physical displacement of accumulated gases, nevertheless the potential contribution of immediate microbial responses warrants additional study. High fluxes were further sustained for some hours by rapid microbial activation as a response to moisture increase. The main driver of N_2_O fluxes was water availability, followed by net mineralization and nitrification rates, and C and N stocks.

On the other hand, a rainfall pulse had a negative impact on N_2_O emissions in sediments from a temperate intermittent river, as its magnitude increased (4 mm vs. 21 mm rainfall) (Arce et al., 2018). The effect of rewetting on N_2_O fluxes was further modulated by the duration of the preceding drying period (6 and 9 weeks). Intense rainfall (21 mm) caused a decrease in N_2_O fluxes, while following modest rainfall (4 mm) emissions increased immediately after the water pulse 24 h later. The decrease of the average fluxes was most likely related to physical trapping preventing gas exchange and displacement during the monitoring period (24 h). Comparing N_2_O pulses immediately after drainage and rewetting, the former pulse was of smaller magnitude (≈ 5 vs. 17 µg N_2_O-N m^−2^ h^−1^ increase in emissions, respectively) when considering the rewetting pulse after the longer preceding drying period under moderate rainfall (Arce et al., 2018; Table 1). These changes in N_2_O emissions stress the importance of considering the previous hydrological history, as biogeochemical responses and emission sources are affected by the sediment’s preceding conditions (e.g., Gómez et al., 2012; Wilson & Baldwin, 2008; Reverey et al., 2018).

In seasonal pond sediments, rewetting of dry sediments after a rainfall event induced a peak of labile organic carbon and inorganic nitrogen and enhanced microbial activity (Fromin et al., 2010). The percentage of N_2_O emission by denitrification remained high (60%) after rewetting and similar to the one measured during the dry period, suggesting a lack of recovery of the physiological ability of the denitrifying community to reduce N_2_O, and an impact of drought on the community structure. Potential nitrification rates also slightly increased. Preceding dry conditions also affect sediment microbial activity. Sediments subject to frequent dry-wet cycles displayed a stronger stimulation of potential microbial activities (denitrification enzyme activity) and higher potential for N_2_O emissions after rewetting compared to sediments undergoing infrequent drying phases. For instance, the potential denitrification rates after rewetting were highest at sites with longer dry periods, namely at the pond edges. The potential denitrification rates triggered by the rewetting pulse were comparable to the maximum rates measured during drying (Fromin et al., 2010).

### Effects of flooding and water level fluctuations on N_2_O emissions in freshwater sediments: aquatic-terrestrial transition zones

Specific regions present in both temporary and perennial systems may experience recurrent water level fluctuations (flooding, water drawdown). These include gravel bars and the parafluvial zone in lotic systems (rivers and streams) and the littoral zones of lentic systems (ponds, lakes and reservoirs), in which potentially large areas of sediments are exposed to the atmosphere during water level fluctuations, and are potential sources of N_2_O. Similarly to the previous sections, the hydrological and O_2_ dynamics will affect N_2_O fluxes through physical mechanisms and microbial processes (Figs. 2 and 3). Rises in the water table and flooding create anoxic conditions, stimulating denitrification and N_2_O production, while water drawdown increases aerobic processes and N_2_O diffusion to the surface. For example, Morse & Bernhardt (2013) found that nitrification contributed significantly more to N_2_O yields in sediments after drainage than denitrification. Water level drawdown and drying of sediments may temporarily increase NH${}_{4}^{+}$ and NO${}_{3}^{-}$ concentrations upon inundation. N losses might be high during initial rewetting and slow down with prolonged inundation (Arce, Sánchez-Montoya & Gómez, 2015; Reverey et al., 2016). Intensified water level fluctuations may also accelerate dissolved organic carbon mineralization, thereby increasing the availability of organic carbon to fuel denitrification (Shi et al., 2020).

High N turnover rates and locally high N_2_O efflux were found in frequently flooded river gravel bars, including co-occurrence of high-rates of both, nitrification and denitrification (Shrestha et al., 2012; Table 1). During drying phases, accumulated or freshly produced NO${}_{3}^{-}$ was quickly denitrified, as suggested by higher potential denitrification rates compared to gross nitrification. The high spatial variability of N_2_O emissions and a highly variable N_2_:N_2_O ratio, arising from the variety of processes involved in the production and consumption of N_2_O, makes it difficult to correlate between fluxes and processes (e.g., potential denitrification). Furthermore, Shrestha et al. (2014) observed that flood pulses promoted a substantial temporary increase in N mineralization on frequently flooded gravel bars (Table 1). The cause of the transient increase in microbial activity was attributed to the input of fresh, highly available organic N (non-structured allochthonous material) and enhanced turnover of previously protected N from aggregate disruption during flood pulses. In turn, an increase in N mineralization stimulated coupled nitrification-denitrification during the drying phase, as oxic-anoxic conditions were favorable for the co-occurrence of both processes (spatially heterogeneous aggregate structure). Thus, areas exposed to short intensive floods with fast-flowing water can be considered as hot spots, and the dry phases after the flood as hot moments of N transformations, which may lead to temporarily high N_2_O emissions. Similarly, McIntyre et al. (2009) observed that in sediments from an intermittent stream, large flood pulses resulting in full saturation cause a slower release of mineralization products, compared to small pulse events that stimulate a rapid cycle of C and N mineralization–immobilization. In sediments subject to 50% saturation, >90% of the mineralized N accumulated within the first 7 d of incubation, compared to only 48% when fully saturated (100% saturation).

In the parafluvial zone, N_2_O emissions were measured along a hydrological gradient covering rarely-flooded and frequently flooded sediments, as well as elevated non-flooded soils in the floodplain (Pinto et al., 2020). Fluxes were higher during the period of higher water level variability, when sediments were subject to frequent drying and rewetting, compared to the desiccation period and post-flood period. Higher fluxes were attributed to a tight coupling between nitrification and denitrification during this period (Table 1). N_2_O emissions were generally higher in sediments exposed to frequent dry-wet cycles, compared to non-flooded and rarely-flooded sediments. Emissions in the frequently flooded sediments were mainly C driven, and the variation in N_2_O fluxes was explained by the quality of the dissolved organic matter pool, which can influence microbial processes such as denitrification (Barnes, Smith & Aiken, 2012).

The microbial use of available organic C is known to be affected by dry-wet cycles. The flow of allochthonous derived C through the microbial community has been tracked in sediments exposed to water level changes (Weise et al., 2016). Terrestrially derived allochthonous organic C was the main source of C under hydrological fluctuations, whereas in permanently dry sediments, the main source of OC originated from C stored in the sediments. Thus, allochthonous OC is more intensively used under dry-wet conditions showing that repeated dry-wet cycles promote OM degradation and increased substrate availability (Weise et al., 2016). Results from these studies indicate that even in perennial systems, certain regions are potential biogeochemical hotspots for GHG emissions during periods of water fluctuation, namely the parafluvial zone, which is a highly active and dynamic area.

The short-term effects of flood pulses on N_2_O fluxes were quantified in artificial riparian marshes along a similar transverse gradient covering dry, alternate dry-wet and permanently flooded sediment conditions (Hernandez & Mitsch, 2006; Table 1). Permanently flooded plots showed the lowest N_2_O fluxes, however, it remains unclear whether this was attributed to higher N_2_:N_2_O ratio due to complete denitrification or to restricted diffusion transport from the presence of standing water. Intermittently flooded sediments and sediments exposed to air showed significantly higher N_2_O emissions during and after the flood pulse than before the pulse. Such pulsing conditions might have enhanced coupled nitrification-denitrification, but also caused suboptimal conditions for denitrification (inhibition of N_2_O reductase by O_2_), decreasing the N_2_:N_2_O ratio. At lower water table, N_2_O emissions were most likely the by-product of nitrification, as seen by the high concentration of NO${}_{3}^{-}$ and low concentration of NH${}_{4}^{+}$. Under absence of standing water, N_2_O diffusion to the atmosphere was unrestricted, increasing N_2_O fluxes. Similarly, N_2_O emissions were detected in exposed sediments during the low water period, but were never detected in flooded lake sediments, as nitrification and NO${}_{3}^{-}$ supply to denitrification were restricted (Kern et al., 1996; Table 1). As air enters the sediment during low water, O_2_ conditions are favorable for OM mineralization, increasing NH${}_{4}^{+}$ and subsequent oxidation by nitrification. Since NO${}_{3}^{-}$ did not accumulate in the pore water of exposed sediments, N_2_O was presumed to derive from coupled nitrification-denitrification.

Other studies in lakes highlight littoral zones as potential N_2_O sources, where drying and wetting cycles are a significant factor controlling denitrification and N_2_O emissions. Denitrification and N_2_O emission rates were found to be higher in the exposed regions and increased with the degree of sediment dryness, whereas in the permanently flooded littoral zone rates were near zero (Huttunen et al., 2003; Akatsuka & Mitamura, 2011; Table 1). High emissions were attributed to enhanced N turnover and nitrification in the aerobic sediment surface, and/or denitrification in anaerobic microsites. Furthermore, potential denitrification rates and potential N_2_O production rates are higher in sediments from littoral zones compared to land soils (Wang et al., 2012; Table 1). Nevertheless, a higher proportion of N_2_O was transformed into N_2_ in the sediments, and attributed to the short supply of NO${}_{\mathrm{x}}^{-}$. If drying events are expected to intensify in the future, these results suggest that sediments from littoral zones have higher potential than soils to emit N_2_O, as NO${}_{3}^{-}$ and O_2_ availability increase in exposed sediment with drying.

Hydroelectric reservoirs experience intensified water level fluctuations due to peak and off-peak electricity demand, creating frequent wetting–drying cycles over potentially large water drawdown areas. In these areas, potential denitrification rates were strongly related to the period of water level rising-falling cycle (Shi et al., 2020). Both denitrification rate and N_2_O fluxes were higher closer to the water edge, where large amounts of N_2_O were produced and emitted at a high rate (Table 1). Water level fluctuations further enhanced hyporheic exchanges, which accelerated organic carbon mineralization, increasing the carbon source supply for denitrification.

Kettle holes have likewise been identified as significant sources of N_2_O (Merbach et al., 2002; Table 1). N_2_O emissions in sediments negatively correlated with the water level along a depositional transect exposed to different drying frequencies, with higher water level slightly decreasing emissions. The legacy of dry-wet cycling is similarly important. Nitrogen loss from these ecosystems is potentially highest in hydrologically less stable sediments (frequently exposed pond margins, desiccated for longer periods), which were depleted in NH${}_{4}^{+}$ during sediment exposed conditions, likely due to nitrification (Reverey et al., 2018; Table 1). The hydrological history (predominantly inundated, intermediate/occasionally dry, and sediments frequently exposed to dry-wet cycles) in kettle holes created distinct microbial habitats, reflected by changes in the microbial community structure. During sediment exposure (higher oxygenation), DNRA potential was higher in sediments less exposed to drought, while potential denitrification was higher in frequently exposed sediments. Nevertheless, denitrification was always higher than DNRA. The nitrification potential increased towards the more frequently exposed sediments as a result of higher oxygenation due to longer exposure to the atmosphere (Reverey et al., 2018). These results highlight the importance of considering the biogeochemical legacy of dry-wet cycles when predicting ecosystem responses to changing conditions (inundation vs. sediment exposure), namely N_2_O emissions.

## Effects of dry-wet cycles on microbial community composition and function

The frequency and duration of dry-wet cycles influence microbial community composition and response, and consequently the biogeochemical processes involved in N_2_O emissions. Repeated dry–wet cycles may result in a permanent change of bacterial community composition and function (Pohlon, Fandino & Marxsen, 2013; Marxsen, Zoppini & Wilczeck, 2010). Cycles of drought and abrupt rewetting may exert long-term effects at the community level, e.g., Gram-negative bacteria (single-layer cell wall) are more susceptible to osmotic stress than fungi and Gram-positive bacteria (thick, strong cell wall). As nitrifiers and most denitrifiers are Gram-negative, the drying-rewetting stress may ultimately affect the biogeochemical capabilities of the different groups, namely the metabolic pathways of nitrification and denitrification (Schimel, Balser & Wallenstein, 2007).

Microbial communities exposed to frequent dry-wet cycles appear to be more tolerant to the drying and rewetting stress (Fierer, Schimel & Holden, 2003; Schimel, Balser & Wallenstein, 2007; Borken & Matzner, 2009; Fromin et al., 2010; Marxsen, Zoppini & Wilczeck, 2010; Peralta, Ludmer & Kent, 2013; Weise et al., 2016). If the community is resistant, resilient and/or functionally redundant, ecosystem process rates may not be altered. It is also possible that large changes in ecosystem process rates are not accompanied by changes in community composition, e.g., due to immediate physiological responses to environmental fluctuations (Koschorreck & Darwich, 2003; Allison & Martiny, 2008). Studies have suggested a certain resistance of denitrifiers and ammonia oxidizers to desiccation in temporary fluvial systems, while significantly changing their microbial activity in response to changes/dry-wet cycles (Peralta, Ludmer & Kent, 2013; Merbt et al., 2016; Arce et al., 2018; Gionchetta et al., 2019; Gionchetta et al., 2020). This points to a decoupling between microbial community structure and functioning (i.e., rates of microbial activities) under varying hydrological conditions, as process rates do not appear to be strongly coupled to changes in abundance and community composition, and extracellular enzyme activity is able to recover to pre-drought levels despite irreversible changes in community structure (Foulquier et al., 2013; Pohlon, Fandino & Marxsen, 2013; Manis et al., 2014).

Overall, functional rates in temporary systems experiencing dry-wet cycles are more dependent on environmental conditions rather than community composition, as microbial community structure is often similar between both phases, while process rates such as denitrification change (Zoppini & Marxsen, 2010; Febria et al., 2015). For example, although the overall microbial community structure may be largely similar between temporary and permanent stream sediments, different taxonomic associations may dominate in temporary (nitrite-oxidizing bacteria) and permanent (methanotrophs) systems (Febria et al., 2015). This suggests that only a part of the community is more responsive to changes in dry-wet cycles. The link between changes in environmental conditions, functional shifts and N_2_O fluxes is currently a knowledge gap.

## Concluding remarks

This synthesis evidences that dry-wet cycles generate hot spots and hot moments of N_2_O production and emission in sediments from a wide array of aquatic habitats. N_2_O emission peaks are observed following sediment rewetting events (rainfall, flooding, and water level fluctuations), as well as after water drainage and/or during sediment desiccation. Moreover and as recently emphasized for CO_2_ emissions, studies suggest that exposed sediments during dry phases can be active spots for N_2_O emissions. Fluxes from exposed sediments are in the range of those observed from terrestrial soils. Nevertheless, the highest N_2_O emission peaks have been measured for the latter so far (Kim et al., 2012, Appendix A).

The general mechanisms behind N_2_O emissions during drying and rewetting are comparable to those of soils, and are mainly related to physical mechanisms and enhanced microbial processing in both lotic and lentic habitats. In a first stage, high instantaneous fluxes are attributed to the release of entrapped N_2_O in the sediment, and the physical processes driving N_2_O emissions are mainly regulated by water fluctuations in the sediment. Following, a period of enhanced microbial activity, driven by increased nutrient availability, sustains higher N_2_O emissions. At early desiccation, upon rewetting or during periods of water level fluctuation, nitrification and denitrification are reported to be strongly stimulated. Higher processing rates and N_2_O fluxes have been mainly observed when conditions are suboptimal for both nitrification and denitrification, and the processes are coupled. These conditions are largely determined by O_2_ availability. Microbial activity and responses to changing conditions are also affected by the magnitude and duration of the dry and wet periods, similarly to those observed in soils.

The similarity between soil and sediments extends to the effects of dry-wet cycling on N_2_O fluxes. This further corroborates the concept proposed by Arce et al. (2019) suggesting that the biogeochemical drivers of sediments are similar to those of soils, and comparable in terms of microbial communities and biogeochemical processes. The reviewed studies support the observation that sediments exposed to dry-wet cycles are hotspots for temporarily high N_2_O emissions, with peak timing and duration depending on the conditions preceding each phase, as previously highlighted for C fluxes in sediments (Marcé et al., 2019). The peak fluxes appear to be of short duration, however, their relevance for global emission estimates has not been quantified as information is scarce. In terrestrial systems, short-lived peak emissions may contribute more than 50% to cumulative and annual emissions, and occur within a smaller number of events (Dorich et al., 2020).

N_2_O emissions from inland open waters remain a major source of uncertainty in global emission estimates (Maavara et al., 2018). Further neglecting the areas alternating between exposed and flooded sediments, as well as N_2_O emissions from dry inland waters, may raise even higher uncertainties as these regions may change on a large scale depending on precipitation and drying events, and water level fluctuations. These conditions are a predictable factor controlling nitrification, denitrification, and N_2_O production and emissions, as redox potential and nitrate concentrations are significantly affected by dry-wet cycles.

### Future research

In the reviewed studies, the experimental design and the different temporal scales between and within studies made it difficult to compare the dynamics of N_2_O fluxes as each phase progresses (wet to dry or dry to wet phase), and hot moments may have been overlooked. Addressing this gap may be of significance when modeling ecosystem responses to dry-wet cycles, as the direction of the fluctuation may influence N_2_O predictions. For instance (in soils), it has been observed that the intensities of N_2_O emission peaks can be higher during the drying phase compared to the wetting (near-saturation) phase, and correlated with the amount of water drained, although with shorter peak duration (Rabot, Hénault & Cousin, 2014). In sediments, the driving role of water fluctuations on N_2_O peak fluxes is evident, and future research should address the temporal development during both drying and rewetting phases in more detail, capturing the rapid changes at early stages. Therefore, efforts should be made to better assess the timing of these events, in order to estimate the relative contribution of hot moments and significance of these peaks to cumulative emissions. This will allow for a more accurate integration of emissions from inland waters. N_2_O uptake in aquatic systems also merits further investigation, as the capacity of aquatic systems to act as sinks may change in the future with changes in net precipitation (Chapuis-Lardy et al., 2007; Kroeze, Bouwman & Slomp, 2007).

The importance of the dry-wet cycling legacy is also recognized, as it shapes the microbial community composition and function. Future research efforts tackling N_2_O emissions during dry-wet cycles should likewise elucidate how environmental changes (i.e., drying and rewetting) shape the network interactions in microbial communities, as well as the functional potentials of the active microbial associations (RNA sequencing and ecological network analysis, e.g., see Fuhrman, 2009; Zhou et al., 2010; Cong et al., 2015). Studies have suggested that some microbial associations are found to alter predictably, and changes in activity under shifting conditions may be anticipated (Fuhrman, 2009). Focusing beyond the abundance of functional groups may prove valuable to develop and improve modeled N_2_O emission estimates further, namely in temporary systems. A comprehensive study should include distinct inland waters exposed to different frequency, intensity and duration of drying and rewetting (perennial, intermittent, ephemeral, and episodic systems; according to definitions in Datry, Bonada & Boulton, 2017). Although it is challenging to predict the timing of events in these systems, this would allow for a broader view on the impacts of frequency and duration of dry-wet cycles on N_2_O emissions. Laboratory incubations, mesocosms and microcosms studies are an asset to understand the evolution and determine the pathways for N_2_O production and emissions during dry-wet cycles (e.g., ^15^N stable isotopic methods). Most studies assume the controlling N_2_O production pathway depending on the predominant redox conditions, and more efforts should be made to determine the relative contribution of each production pathway to N_2_O emissions during each phase (wet to dry or dry to wet). Nevertheless, these studies tend to oversimplify ecosystem interactions, namely between surface and subsurface sediments, potentially excluding significant N_2_O input from deeper sediments (Lansdown et al., 2015; Welter & Fisher, 2016). Despite the inherent difficulties, experimental studies should be complemented by field studies covering natural hydrological gradients within and among ecosystems. Only connective knowledge can help to better understand the extremely dynamic behavior of N_2_O in freshwater sediments under dry-wet stress.

## Additional Information and Declarations

### Data Availability

The following information was supplied regarding data availability: This is a review article; there are no raw data.

## References

[ref-1] Akatsuka T, Mitamura O (2011). Response of denitrification rate associated with wetting and drying cycles in a littoral wetland area of Lake Biwa, Japan. Limnology.

[ref-2] Allison S, Martiny J (2008). Resistance, resilience, and redundancy in microbial communities. Proceedings of the National Academy of Sciences of the United States of America.

[ref-3] Amalfitano S, Fazi S, Zoppini A, Barra Caracciolo A, Grenni P, Puddu A (2008). Responses of benthic bacteria to experimental drying in sediments from Mediterranean temporary rivers. Microbial Ecology.

[ref-4] Arce MI, Mendoza-Lera C, Almagro M, Catalán N, Romaní AM, Martí E, Gómez R, Bernal S, Foulquier A, Mutz M, Marcé R, Zoppini A, Gionchetta G, Weigelhofer G, Del Campo R, Robinson CT, Gilmer A, Rulik M, Obrador B, Shumilova O, Zlatanović S, Arnon S, Baldrian P, Singer G, Datry T, Skoulikidis N, Tietjen B, Von Schiller D (2019). A conceptual framework for understanding the biogeochemistry of dry riverbeds through the lens of soil science. Earth-Science Reviews.

[ref-5] Arce MI, Sánchez-Montoya MM, Gómez R (2015). Nitrogen processing following experimental sediment rewetting in isolated pools in an agricultural stream of a semiarid region. Ecological Engineering.

[ref-6] Arce MI, Sánchez-Montoya MM, Vidal-Abarca MR, Suárez ML, Gómez R (2014). Implications of flow intermittency on sediment nitrogen availability and processing rates in a Mediterranean headwater stream. Aquatic Sciences.

[ref-7] Arce MI, Von Schiller D, Bengtsson MM, Hinze C, Jung H, Eloy Alves RJ, Urich T, Singer G (2018). Drying and rainfall shape the structure and functioning of nitrifying microbial communities in riverbed sediments. Frontiers in Microbiology.

[ref-8] Aulakh MS, Doran JW, Mosier AR, Stewart BA (1992). Soil denitrification—significance, measurement, and effects of management. Advances in Soil Science.

[ref-9] Austin BJ, Strauss EA (2011). Nitrification and denitrification response to varying periods of desiccation and inundation in a western Kansas stream. Hydrobiologia.

[ref-10] Baggs EM (2011). Soil microbial sources of nitrous oxide: recent advances in knowledge, emerging challenges and future direction. Current Opinion in Environmental Sustainability.

[ref-11] Baldwin DS, Mitchell AM (2000). The effects of drying and re-flooding on the sediment and soil nutrient dynamics of lowland river-floodplain systems: a synthesis. Regulated Rivers: Research and Management.

[ref-12] Barnes R, Smith R, Aiken GR (2012). Linkages between denitrification and dissolved organic matter quality, Boulder Creek watershed, Colorado. Journal of Geophysical Research: Biogeosciences.

[ref-13] Bateman E, Baggs E (2005). Contributions of nitrification and denitrification to N_2_O emissions from soils at different water-filled pore space. Biology and Fertility of Soils.

[ref-14] Beare MH, Gregorich EG, St-Georges P (2009). Compaction effects on CO_2_ and N_2_O production during drying and rewetting of soil. Soil Biology and Biochemistry.

[ref-15] Birch HF (1958). The effect of soil drying on humus decomposition and nitrogen availability. Plant Soil.

[ref-16] Borken W, Matzner E (2009). Reappraisal of drying and wetting effects on C and N mineralization and fluxes in soils. Global Change Biology.

[ref-17] Castellano MJ, Lewis DB, Andrews DM, Mcdaniel MD, Lin, Henry (2012). Coupling biogeochemistry and hydropedology to advance carbon and nitrogen cycling science. Hydropedology: synergistic integration of soil science and hydrology.

[ref-18] Catalan N, Von Schiller D, Marcé R, Koschorreck M, Gómez-Gener L, Obrador B (2014). Carbon dioxide efflux during the flooding phase of temporary ponds. Limnetica.

[ref-19] Chapuis-Lardy L, Wrage N, Metay A, Chottes J-L, Bernoux M (2007). Soils, a sink N2O? A review. Global Change Biology.

[ref-20] Cong J, Yang Y, Liu X, Lu H, Liu X, Zhou J, Li D, Yin H, Ding J, Zhang Y (2015). Analyses of soil microbial community compositions and functional genes reveal potential consequences of natural forest succession. Scientific Reports.

[ref-21] Congreves KA, Wagner-Riddle C, Si BC, Clough TJ (2018). Nitrous oxide emissions and biogeochemical responses to soil freezing-thawing and drying-wetting. Soil Biology and Biochemistry.

[ref-22] Datry T, Bonada N, Boulton AJ, Datry T, Bonada N, Boulton AJ (2017). General introduction. Intermittent rivers and ephemeral streams: ecology and management.

[ref-23] Datry T, Larned ST, Tockner K (2014). Intermittent rivers: a challenge for freshwater ecology. BioScience.

[ref-24] Denef K, Six J, Bossuyt H, Frey SD, Elliott ET, Merckx R, Paustian K (2001). Influence of dry–wet cycles on the interrelationship between aggregate, particulate organic matter, and microbial community dynamics. Soil Biology and Biochemistry.

[ref-25] Dorich CD, Conant RT, Albanito F, Butterbach-Bahl K, Grace P, Scheer C, Snow VO, Vogeler I, Van der Weerden TJ (2020). Improving N2O emission estimates with the global N2O database. Current Opinion in Environmental Sustainability.

[ref-26] Febria CM, Hosen JD, Crump BC, Palmer MA, Williams DD (2015). Microbial responses to changes in flow status in temporary headwater streams: across-system comparison. Frontiers in Microbiology.

[ref-27] Fierer N, Schimel JP (2003). A proposed mechanism for the pulse in carbon dioxide production commonly observed following the rapid rewetting of a dry soil. Soil Science Society of America Journal.

[ref-28] Fierer N, Schimel JP, Holden PA (2003). Influence of drying-rewetting frequency on soil bacterial community structure. Microbial Ecology.

[ref-29] Foulquier A, Artigas J, Pesce S, Datry T (2015). Drying responses of microbial litter decomposition and associated fungal and bacterial communities are not affected by emersion frequency. Freshwater Science.

[ref-30] Foulquier A, Volat B, Neyra M, Bornette G, Montuelle B (2013). Long-term impact of hydrological regime on structure and functions of microbial communities in riverine wetland sediments. FEMS Microbiology Ecology.

[ref-31] Fromin N, Pinay G, Montuelle B, Landais D, Ourcival JM, Joffre R, Lensi R (2010). Impact of seasonal sediment desiccation and rewetting on microbial processes involved in greenhouse gas emissions. Ecohydrology.

[ref-32] Fuhrman J (2009). Microbial community structure and its functional implications. Nature.

[ref-33] Gallo EL, Lohse KA, Ferlin CM, Meixner T, Brooks PD (2014). Physical and biological controls on trace gas fluxes in semi-arid urban ephemeral waterways. Biogeochemistry.

[ref-34] Giles M, Morley N, Baggs E, Daniell T (2012). Soil nitrate reducing processes—drivers, mechanisms for spatial variation, and significance for nitrous oxide production. Frontiers in Microbiology.

[ref-35] Gionchetta G, Oliva F, Romaní AM, Bañeras L (2020). Hydrological variations shape diversity and functional responses of streambed microbes. Science of The Total Environment.

[ref-36] Gionchetta G, Romaní AM, Oliva F, Artigas J (2019). Distinct responses from bacterial, archaeal and fungal streambed communities to severe hydrological disturbances. Scientific Reports.

[ref-37] Gómez R, Arce MI, Sánchez JJ, Sanchez-Montoya MM (2012). The effects of drying on sediment nitrogen content in a Mediterranean intermittent stream: a microcosms study. Hydrobiologia.

[ref-38] Gómez-Gener L, Obrador B, Marcé R, Acuña V, Catalán N, Casas-Ruiz JP, Sabater S, Muñoz I, Von Schiller D (2016). When water vanishes: magnitude and regulation of carbon dioxide emissions from dry temporary streams. Ecosystems.

[ref-39] Gómez-Gener L, Obrador B, Von Schiller D, Marcé R, Casas-Ruiz JP, Proia L, Acuña V, Catalán N, Muñoz I, Koschorreck M (2015). Hot spots for carbon emissions from Mediterranean fluvial networks during summer drought. Biogeochemistry.

[ref-40] Gownaris NJ, Rountos KJ, Kaufman L, Kolding J, Lwiza KMM, Pikitch EK (2018). Water level fluctuations and the ecosystem functioning of lakes. Journal of Great Lakes Research.

[ref-41] Hernandez ME, Mitsch WJ (2006). Influence of hydrologic pulses, flooding frequency, and vegetation on nitrous oxide emissions from created riparian marshes. Wetlands.

[ref-42] Holgerson M, Raymond P (2016). Large contribution to inland water CO_2_ and CH_4_ emissions from very small ponds. Nature Geoscience.

[ref-43] Huttunen J, Sari J, Jukka A, Larmola T, Hammar T, Silvola J, Martikainen P (2003). Nitrous oxide flux to the atmosphere from the littoral zone of a boreal lake. Journal of Geophysical Research-Atmospheres.

[ref-44] Jin H, Yoon TK, Lee S-H, Kang H, Im J, Park J-H (2016). Enhanced greenhouse gas emission from exposed sediments along a hydroelectric reservoir during an extreme drought event. Environmental Research Letters.

[ref-45] Jørgensen BB, Revsbech NP (1985). Diffusive boundary-layers and the oxygen-uptake of sediments and detritus. Limnology and Oceanography.

[ref-46] Kern J, Darwich A, Furch K, Junk WJ (1996). Seasonal denitrification in flooded and exposed sediments from the Amazon floodplain at Lago Camaleão. Microbial Ecology.

[ref-47] Kim DG, Vargas R, Bond-Lamberty B, Turetsky MR (2012). Effects of soil rewetting and thawing on soil gas fluxes: a review of current literature and suggestions for future research. Biogeosciences.

[ref-48] Knowles R (1982). Denitrification. Microbiological Reviews.

[ref-49] Koschorreck M (2005). Nitrogen turnover in drying sediments of an Amazon floodplain lake. Microbial Ecology.

[ref-50] Koschorreck M, Darwich A (2003). Nitrogen dynamics in seasonally flooded soils in the Amazon floodplain. Wetlands Ecology and Management.

[ref-51] Kosten S, Van den Berg S, Mendonça R, Paranaíba JR, Roland F, Sobek S, Van Den Hoek J, Barros N (2018). Extreme drought boosts CO2 and CH4 emissions from reservoir drawdown areas. Inland Waters.

[ref-52] Kroeze C, Bouwman L, Slomp CP (2007). Sinks for nitrous oxide at the earth’s surface. Greenhouse gas sinks.

[ref-53] Langhans SD, Tockner K (2006). The role of timing, duration, and frequency of inundation in controlling leaf litter decomposition in a river-floodplain ecosystem (Tagliamento, NE Italy). Oecologia.

[ref-54] Lansdown K, Heppell CM, Trimmer M, Binley A, Heathwaite AL, Byrne P, Zhang H (2015). The interplay between transport and reaction rates as controls on nitrate attenuation in permeable, streambed sediments. Journal of Geophysical Research: Biogeosciences.

[ref-55] Larned ST, Datry T, Robinson CT (2007). Invertebrate and microbial responses to inundation in an ephemeral river reach in New Zealand: effects of preceding dry periods. Aquatic Sciences.

[ref-56] Leigh C, Boulton AJ, Courtwright JL, Fritz K, May CL, Walker RH, Datry T (2015). Ecological research and management of intermittent rivers: an historical review and future directions. Freshwater Biology.

[ref-57] Leira M, Cantonati M (2015). Effects of water-level fluctuations on lakes: an annotated bibliography. Hydrobiologia.

[ref-58] Maavara T, Lauerwald R, Laruelle G, Akbarzadeh Z, Bouskill N, Van Cappellen P, Regnier P (2018). Nitrous oxide emissions from inland waters: are IPCC estimates too high?. Global Change Biology.

[ref-59] Manis E, Royer TV, Johnson LT, Leff LG (2014). Denitrification in agriculturally impacted streams: seasonal changes in structure and function of the bacterial community. PLOS ONE.

[ref-60] Marcé R, Obrador B, Gómez-Gener L, Catalán N, Koschorreck M, Arce M, Singer G, Von Schiller D (2019). Emissions from dry inland waters are a blind spot in the global carbon cycle. Earth-Science Reviews.

[ref-61] Marxsen J, Zoppini A, Wilczeck S (2010). Microbial communities in streambed sediments recovering from desiccation. FEMS Microbiology Ecology.

[ref-62] McClain ME, Boyer EW, Dent CL, Gergel SE, Grimm NB, Groffman PM, Hart SC, Harvey JW, Johnston CA, Mayorga E, McDowell WH, Pinay G (2003). Biogeochemical hot spots and hotmoments at the interface of terrestrial and aquatic ecosystems. Ecosystems.

[ref-63] McIntyre RES, Adams MA, Ford DJ, Grierson PF (2009). Rewetting and litter addition influence mineralization and microbial communities in soils from a semi-arid intermittent stream. Soil Biology and Biochemistry.

[ref-64] Merbach W, Kalettka T, Rudat C, Augustin J, Broll G, Merbach W, Pfeiffer EM (2002). Trace gas emissions from riparian areas of small eutrophic inland waters in Northeast-Germany. Wetlands in Central Europe.

[ref-65] Merbt SN, Proia L, Prosser JI, Martí E, Casamayor EO, Von Schiller D (2016). Stream drying drives microbial ammonia oxidation and first-flush nitrate export. Ecology.

[ref-66] Morse JL, Bernhardt ES (2013). Using 15N tracers to estimate N2O and N2 emissions from nitrification and denitrification in coastal plain wetlands under contrasting land-uses. Soil Biology & Biochemistry.

[ref-67] Obrador B, Von Schiller D, Marcé R, Gómez-Gener L, Koschorreck M, Borrego C, Catalán N (2018). Dry habitats sustain high CO_2_ emissions from temporary ponds across seasons. Scientific Reports.

[ref-68] Peralta AL, Ludmer S, Kent AD (2013). Hydrologic history influences microbial community composition and nitrogen cycling under experimental drying/wetting treatments. Soil Biology and Biochemistry.

[ref-69] Pinto RMS, Weigelhofer G, Diaz-Pines E, Brito AG, Zechmeister-Boltenstern S, Hein T (2020). River-floodplain restoration and hydrological effects on GHG emissions: Biogeochemical dynamics in the parafluvial zone. Science of The Total Environment.

[ref-70] Pohlon E, Fandino AO, Marxsen J (2013). Bacterial community composition and extracellular enzyme activity in temperate streambed sediment during drying and rewetting. PLOS ONE.

[ref-71] Quick AM, Reeder WJ, Farrell TB, Tonina D, Feris KP, Benner SG (2019). Nitrous oxide from streams and rivers: A review of primary biogeochemical pathways and environmental variables. Earth-Science Reviews.

[ref-72] Rabot E, Hénault C, Cousin I (2014). Temporal variability of nitrous oxide emissions by soils as affected by hydric history. Soil Science Society of America Journal.

[ref-73] Reverey F, Ganzert L, Lischeid G, Ulrich A, Premke K, Grossart H-P (2018). Dry-wet cycles of kettle hole sediments leave a microbial and biogeochemical legacy. Science of The Total Environment.

[ref-74] Reverey F, Grossart H-P, Premke K, Lischeid G (2016). Carbon and nutrient cycling in kettle hole sediments depending on hydrological dynamics: a review. Hydrobiologia.

[ref-75] Schimel J, Balser TC, Wallenstein M (2007). Microbial stress-response physiology and its implications for ecosystem function. Ecology.

[ref-76] Seitzinger S, Harrison JA, Bohlke JK, Bouwman AF, Lowrance R, Peterson B, Tobias C, Van Drecht G (2006). Denitrification across landscapes and waterscapes: a synthesis. Ecological Applications.

[ref-77] Seneviratne SI, Nicholls N, Easterling D, Goodess CM, Kanae S, Kossin J, Luo Y, Marengo J, McInnes K, Rahimi M, Reichstein M, Sorteberg A, Vera C, Zhang X (2012). Changes in climate extremes and their impacts on the natural physical environment. Managing the risks of extreme events and disasters to advance climate change adaptation. A special report of working Groups I and II of the Intergovernmental Panel on Climate Change (IPCC).

[ref-78] Shi W, Chen Q, Zhang J, Zheng F, Liu D, Yi Q, Chen Y (2020). Enhanced riparian denitrification in reservoirs following hydropower production. Journal of Hydrology.

[ref-79] Shrestha J, Niklaus PA, Frossard E, Samaritani E, Huber B, Barnard RL, Schleppi P, Tockner K, Luster J (2012). Soil nitrogen dynamics in a river floodplain mosaic. Journal of Environmental Quality.

[ref-80] Shrestha J, Niklaus PA, Pasquale N, Huber B, Barnard RL, Frossard E, Schleppi P, Tockner K, Luster J (2014). Flood pulses control soil nitrogen cycling in a dynamic river floodplain. Geoderma.

[ref-81] Soued C, Giorgio PA, Maranger R (2015). Nitrous oxide sinks and emissions in boreal aquatic networks in Quebec, Canada. Nature Geoscience.

[ref-82] Stein LY, Klotz MG (2016). Primer: the nitrogen cycle. Current Biology.

[ref-83] Steward AL, Von Schiller D, Tockner K, Marshall JC, Bunn SE (2012). When the river runs dry: human and ecological values of dry riverbeds. Frontiers in Ecology and the Environment.

[ref-84] Syakila A, Kroeze C, Slomp CP (2010). Neglecting sinks for N2O at the earth’s surface: does it matter?. Journal of Integrative Environmental Sciences.

[ref-85] Von Schiller D, Bernal S, Dahm CN, Martí E, Datry T, Bonada N, Boulton AJ (2017). Chapter 3.2—Nutrient and Organic Matter Dynamics in Intermittent Rivers and Ephemeral Streams. Intermittent rivers and ephemeral streams: ecology and management.

[ref-86] Von Schiller D, Datry T, Corti R, Foulquier A, Tockner K, Marcé R, García-Baquero G, Odriozola I, Obrador B, Elosegi A, Mendoza-Lera C, Gessner M, Stubbington R, Albariño R, Allen D, Altermatt F, Arce M, Banas D, Zoppini A (2019). Sediment respiration pulses in intermittent rivers and ephemeral streams. Global Biogeochemical Cycles.

[ref-87] Von Schiller D, Marcé R, Obrador B, Gómez-Gener L, Casas-Ruiz JP, Acuña V, Koschorreck M (2014). Carbon dioxide emissions from dry watercourses. Inland Waters.

[ref-88] Wang C, Zhu G, Wang Y, Wang S, Yin C (2012). Nitrous oxide reductase gene (nosZ) and N2O reduction along the littoral gradient of a eutrophic freshwater lake. Journal of Environmental Sciences.

[ref-89] Weise L, Ulrich A, Moreano M, Gessler A, Kayler ZE, Steger K, Zeller B, Rudolph K, Knezevic-Jaric J, Premke K (2016). Desiccation and rewetting of lake sediments causes enhanced carbon turnover and shifts in microbial community composition. FEMS Micobiology Ecology.

[ref-90] Welter JR, Fisher SR (2016). The influence of storm characteristics on hydrologic connectivity in intermittent channel networks: implications for nitrogen transport and denitrification. Freshwater Biology.

[ref-91] Wilson JS, Baldwin DS (2008). Exploring the ‘Birch effect’ in reservoir sediments: influence of inundation history on aerobic nutrient release. Chemistry and Ecology.

[ref-92] Zaman M, Chang SX (2004). Substrate type, temperature, and moisture content affect gross and net N mineralization and nitrification rates in agroforestry systems. Biology and Fertility of Soils.

[ref-93] Zhou J, Deng Y, Luo F, He Z, Tu Q, Zhi X (2010). Functional molecular ecological networks. mBio.

[ref-94] Zoppini A, Marxsen J, Shukla G, Varma A (2010). Importance of extracellular enzymes for biogeochemical processes in temporary river sediments during fluctuating dry–wet conditions. Soil enzymology. Soil biology.

